# Effect of tetramethylammonium hydroxide/isopropyl alcohol wet etching on geometry and surface roughness of silicon nanowires fabricated by AFM lithography

**DOI:** 10.3762/bjnano.7.138

**Published:** 2016-10-17

**Authors:** Siti Noorhaniah Yusoh, Khatijah Aisha Yaacob

**Affiliations:** 1 School of Materials and Mineral Resources Engineering, Engineering Campus University Sains Malaysia, Seri Ampangan, 14300 Nibong Tebal, Penang, Malaysia

**Keywords:** AFM lithography, isopropyl alcohol, silicon nanowires, tetramethylammonium hydroxide, wet etching

## Abstract

The optimization of etchant parameters in wet etching plays an important role in the fabrication of semiconductor devices. Wet etching of tetramethylammonium hydroxide (TMAH)/isopropyl alcohol (IPA) on silicon nanowires fabricated by AFM lithography is studied herein. TMAH (25 wt %) with different IPA concentrations (0, 10, 20, and 30 vol %) and etching time durations (30, 40, and 50 s) were investigated. The relationships between etching depth and width, and etching rate and surface roughness of silicon nanowires were characterized in detail using atomic force microscopy (AFM). The obtained results indicate that increased IPA concentration in TMAH produced greater width of the silicon nanowires with a smooth surface. It was also observed that the use of a longer etching time causes more unmasked silicon layers to be removed. Importantly, throughout this study, wet etching with optimized parameters can be applied in the design of the devices with excellent performance for many applications.

## Introduction

The fabrication of semiconductor devices on silicon-on-insulator (SOI) wafers has recently become popular. Devices necessary for meeting the requirements of SOI applications have been developed. The geometry and surface roughness of the devices are the factors that must be improved in order to upgrade the possible device performance in many fields, such as biomedical applications. The etching process has been studied by many researchers in order to achieve the best performance in the design of semiconductor devices, according to the particular device application. Etching is a complementary process for the top-down fabrication process. Etching processes can be classified as belonging either to the dry or the wet etching process type. The dry etching technique is itself divided into three types: reactive ion etching (RIE) [1–2], sputter etching [3], and vapour phase etching [4]. On the other hand, wet etching is the simplest etching technology and works very well for etching thin films on substrates. Additionally, it can also be used to etch the substrate itself. Wet etching can be either isotropic or anisotropic, depending on the silicon wafer orientation and the type of etchant being used [5–6]. In isotropic etching, the etchant removes the material uniformly in all directions [7], whereas in anisotropic etching, the material is removed uniformly in the vertical direction only. Anisotropic wet etching is mostly used to fabricate simple microstructures and nanostructures on a single crystal SOI wafer [8].

Tetramethylammonium hydroxide (TMAH) [9–11], potassium hydroxide (KOH) [12], sodium hydroxide (NaOH) [13], ethylenediamine-pyrocatechol (EDP) [14–15] and hydrazine/water [14] etchants can be used to remove a single crystal silicon layer. However, certain etchants, such as EDP and hydrazine/water, are not preferable because of their toxicity, instability and difficulty of handling. Sodium hydroxide is rarely used, unlike the potassium hydroxide solution that has become a popular anisotropic etchant because of its good etching performance and lack of toxicity. However, KOH is not CMOS compatible because of the mobile K^+^ ion contamination [16]. By contrast, TMAH has attracted the interest of researchers because of its simple handling and CMOS compatibility.

According to Merlos et al. [16], a smooth surface, free of hillocks can be obtained by using TMAH as an etchant with a concentration greater than 25 wt %. Hutagalung and Lew [17] used 25 wt % of TMAH at 65 °C for 30 s to remove unmasked silicon layers. Then, an improvement in wet etching was obtained by adding isopropyl alcohol (IPA) to a KOH- or TMAH-based solution to enhance the smoothness of the silicon surface [10,12,16,18–19]. Based on these studies, it was claimed that IPA is an effective admixture for the improvement of surface smoothness.

Recently, there have been a few studies on the TMAH/IPA anisotropic etching, but the studies have not investigated the etched product. In this paper, we studied TMAH/IPA wet etching for the fabrication of an array of silicon nanowire patterned by AFM lithography on an SOI wafer. We investigate the relationship between the etching depth and width, and etching rate and surface roughness using TMAH with different IPA concentrations at a constant etching time and also different etching times at a constant IPA concentration. Silicon nanowires with suitable geometrical features and surface are important for obtaining a semiconductor device with excellent performance depending on the device application [20–21].

## Results and Discussion

### Fabrication of silicon nanowires

**Figure 1:**
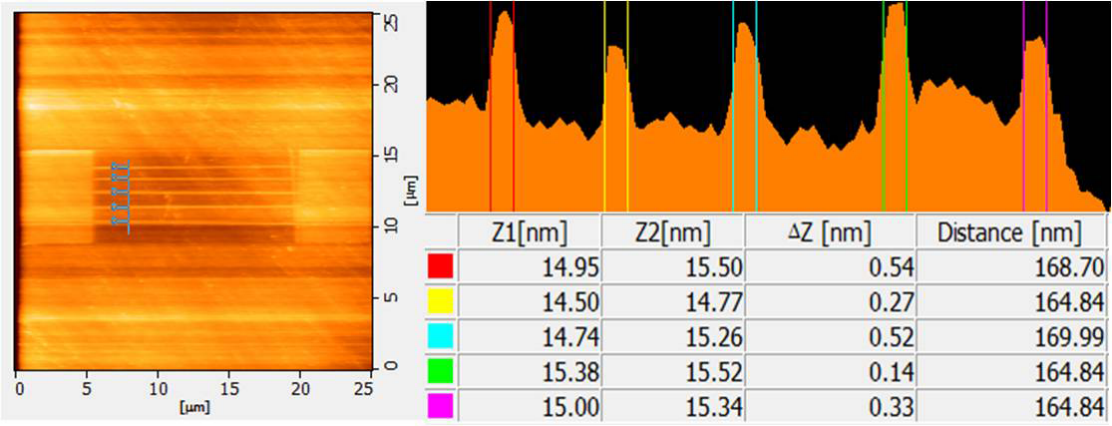
Pattern of silicon oxide nanowire with left and right square pads prior to etching.

In this study, AFM lithography was used to fabricate horizontal silicon nanowires on an SOI wafer. AFM lithography is a top-down approach that starts from large units and proceeds to small units [22–23]. This approach is a well-known method for the fabrication of semiconductor devices in micro and nanoscale structures [24–25]. A biased AFM tip is operated under ambient conditions to oxidize the silicon surface locally and form an oxide mask [26]. Several parameters, such as applied voltage, writing speed and humidity, play important roles in the patterning of the oxide mask [27]. This study adopted parameters reported by Yusoh and Yaacob [27]. They found that use of a contact mode AFM tip coated with Au with 9 V of applied voltage and 0.3 µm/s writing speed at 55–65% relative humidity could produce a thin oxide mask layer with stable and continuous structure on SOI that functioned well as a mask in the subsequent wet chemical etching process. Figure 1 shows that a thin layer oxide mask was patterned into five lines with a width of 165–169 nm, height of 4 nm and gaps of 730 nm between the lines. The square-shaped pads with dimensions of 5 × 5 µm were fabricated on the left and right sides of the silicon nanowire array. Later, these patterns were etched using 25 wt % TMAH with different IPA concentrations. The results were then analysed in terms of etching depth, width, etching rate and surface roughness of the silicon nanowires.

### Etching depth and width analysis

**Figure 2:**
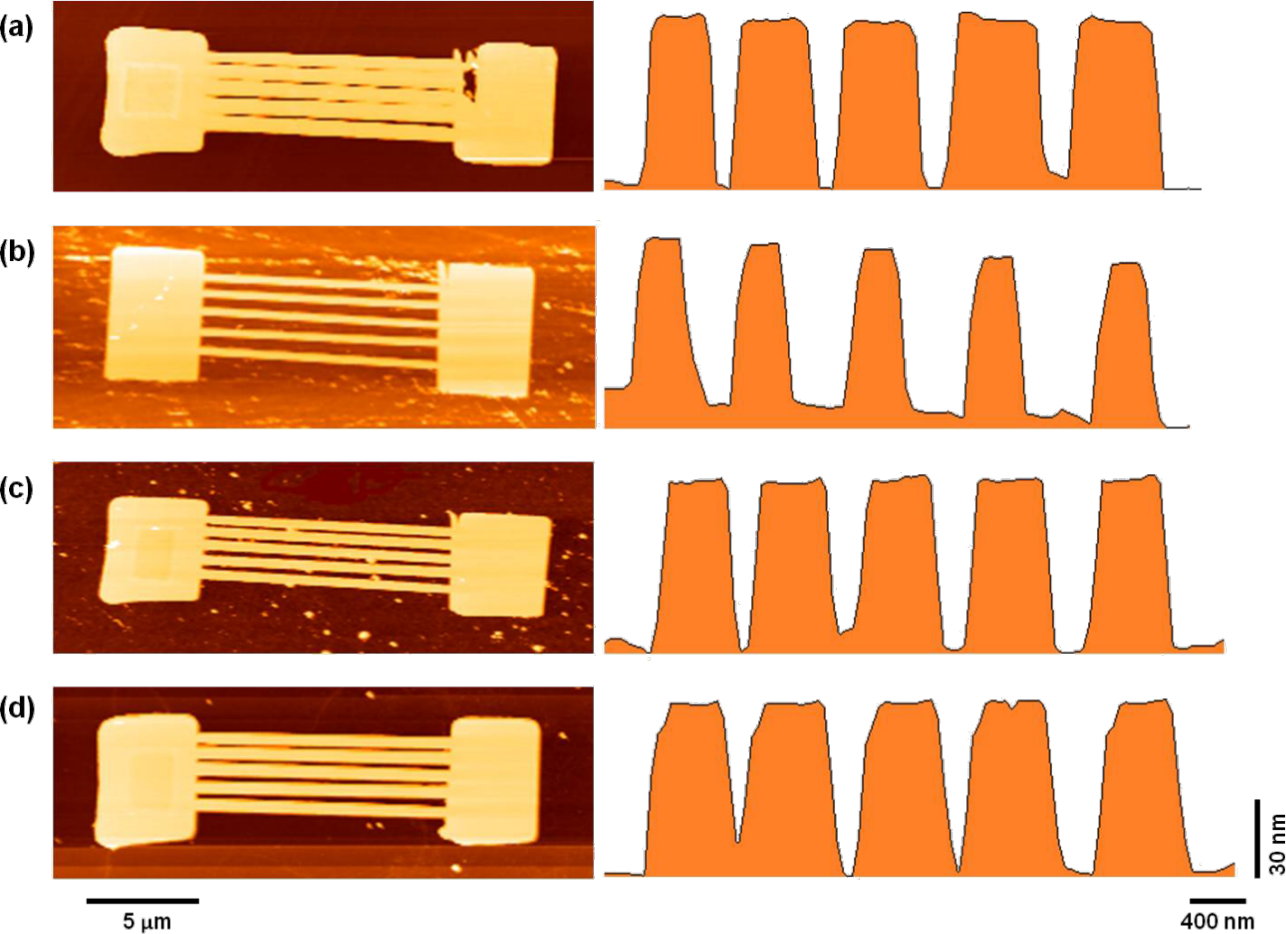
AFM images of silicon nanowire after wet etching using (a) 25 wt % TMAH (b) 25 wt % TMAH with 10 vol % IPA (c) 25 wt % TMAH with 20 vol % IPA (d) 25 wt% TMAH with 30 vol % IPA at 65 °C for 30 s.

The AFM image profile presented in Figure 2 shows that silicon nanowires with trapezoidal cross-section were produced by etching using 25 wt % TMAH without and with IPA (10, 20, 30 vol %). The obtained silicon nanowires are free of hillocks or micropyramids. Both the width and gap dimensions vary based on the concentration of the added IPA. Further addition of IPA into TMAH produced wider silicon nanowires, consequently decreasing the gap between the nanowires.

**Figure 3:**
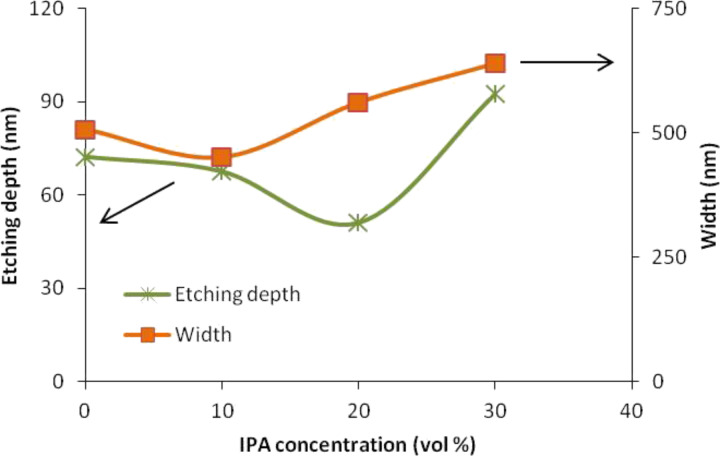
Relation of etching depth and width of a silicon nanowire at different IPA concentrations.

The unmasked silicon layers were uniformly removed at the depth of 72.42 nm without IPA (0 vol %) and 67.68 nm, 51.19 nm, and 92.56 nm with IPA (10 vol %, 20 vol % and 30 vol %, respectively). The dimension of the removed unmasked silicon layers is also known as the etching depth and the remaining silicon layers represent the thickness or height of the fabricated silicon nanowire. Figure 3 shows that the etching depth decreases with the addition of 10 and 20 vol % IPA but then increases at 30 vol %. This trend was the same as that found in the work of Rola and Zubel [28] who explained that only a small amounts of unmasked silicon layers were removed at 20 vol % due to the slow etching rate at that IPA concentration, and more unmasked silicon layers were removed because of the fast etching rate occurring for 30 vol % IPA concentrations. Although more unmasked silicon layers were removed at 30 vol %, the produced silicon nanowires are large and broad compared to other cases. This finding is observed because the different IPA concentrations behave differently to different planes; thus, the etching depth does not influence the width of silicon nanowire. The smallest silicon nanowire widths were produced when etched with 10 vol % IPA. This result led to the repetition of the experiment, but at different etching times with a constant concentration of IPA (10 vol %) and temperature (65 °C). We assumed that the width of the silicon nanowire would be smaller as the etching time was prolonged.

**Figure 4:**
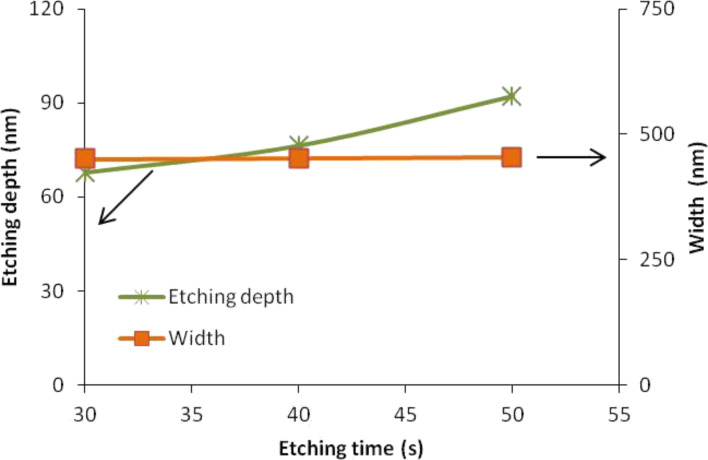
Relationships of etching depth and width of a silicon nanowire at different etching times.

The obtained results show that the widths of the silicon nanowire (450.69, 452.35 and 454.19 nm) do not differ considerably at different etching times (Figure 4). However, the etching depths increased with increasing etching time. The unmasked silicon layer is nearly completely removed in 50 s for 92.3 nm depth out of the 100 nm thickness of the Si layer in the SOI wafer. This observation explained that the exposure of etching time would not result in large changes of the width but will obviously change the height of the silicon nanowire. The longer exposure of etching time will produce thick and tall silicon nanowires, whereas shorter exposure of etching time will produce thin and short silicon nanowires.

**Figure 5:**
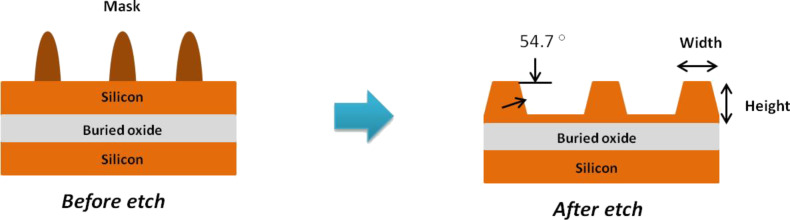
Cross-section of silicon nanowires before and after etching.

**Figure 6:**
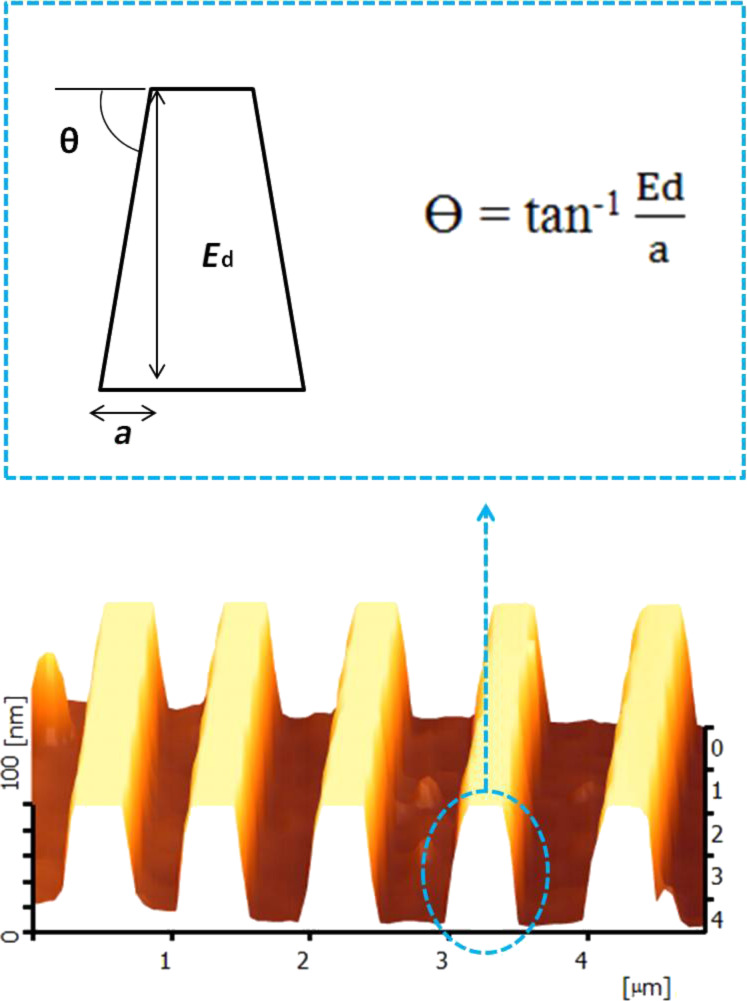
Calculation of silicon nanowire wall angle after etching; *E*_d_ represents the etching depth and *a* is the adjacent segment measured by AFM.

The standard anisotropic etching of the silicon (100) wafer produced a V-groove with a wall angle of 54.7° as shown in Figure 5 [29–30]. However, in this study, we obtained different wall angle values for different IPA concentration conditions. The different concentrations of IPA in TMAH and different etching times produced different widths as well as the etching depth that affect the wall angle values. The calculation of wall angle for silicon nanowires is illustrated in Figure 6, where *E*_d_ is etching depth, and *a* is the adjacent segment that can be measured by using AFM.

**Figure 7:**
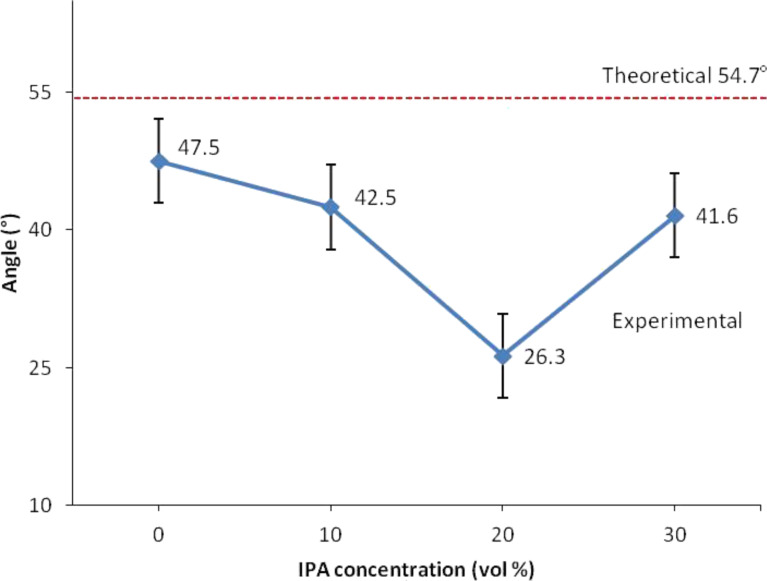
Wall angles obtained at different concentrations of IPA at constant etching time.

**Figure 8:**
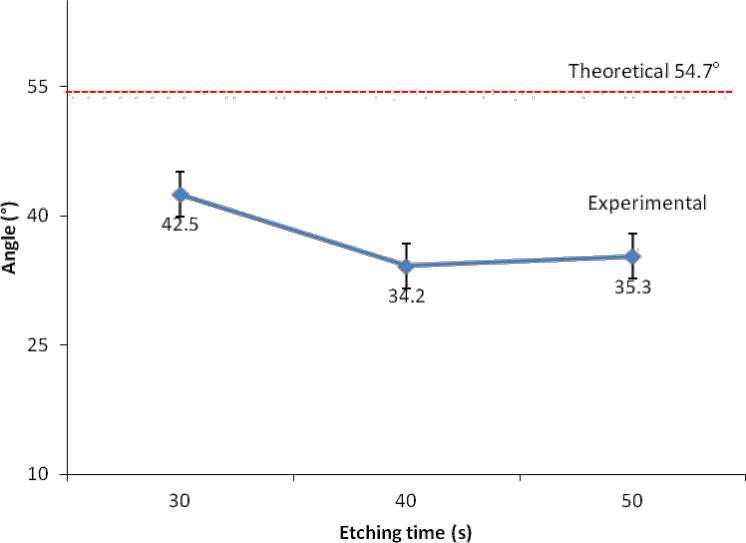
Wall angles obtained at a constant IPA concentration with different etching times.

The angles for each case are depicted in Figure 7 and Figure 8. Figure 7 shows that the plot of the angle trend is similar to the etching depth plot in Figure 3, where the 20 vol % IPA concentration resulted in a small angle value due to the slow etching rate. It is known that a slow etching rate will cause only a small amount of unmasked silicon layers to be removed, therefore producing shorter silicon nanowires. The value of the wall angle becomes small when the height and width of the silicon nanowire are small and large, respectively. However, in Figure 8, the plot of the angle of silicon nanowires etched with a constant concentration of IPA at different etching times shows a trend that is different for the trend of the etching depth plot (Figure 4). This is because the etching depths at different etching times are not similar; this can influence the wall angle measurement even though the measured widths are nearly the same. The etching depth for the etching time of 30 s is smaller than those for the etching times of 40 and 50 s, resulting in wall angles of 42.5°, 34.2° and 35.5°, respectively.

### Etching rate and roughness analysis

**Figure 9:**
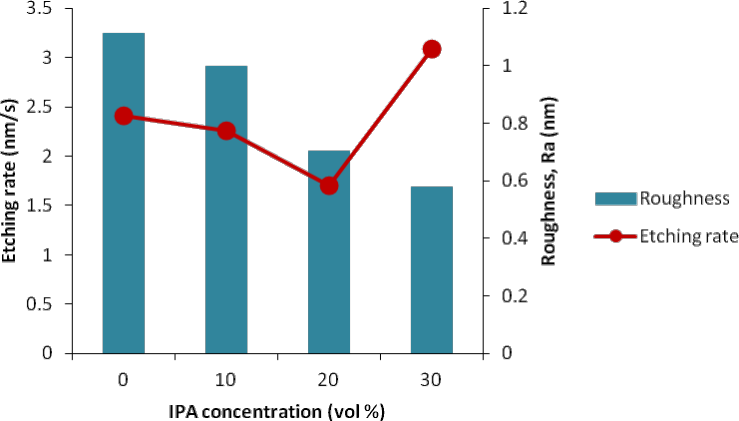
The relationship of the etching rate and surface roughness at different IPA concentrations.

**Figure 10:**
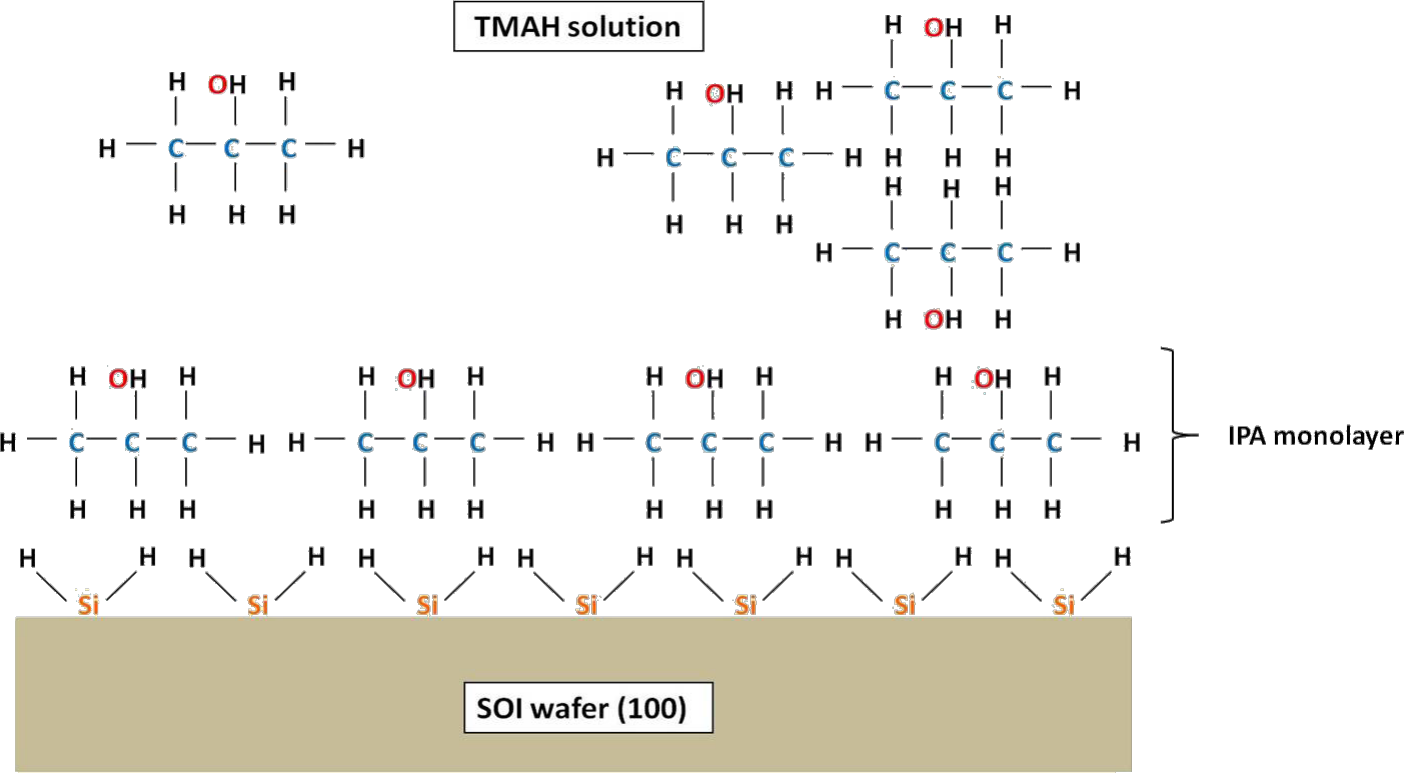
Formation of an IPA monolayer due to a high concentration of IPA in TMAH, resulting in a slow etching rate.

**Figure 11:**
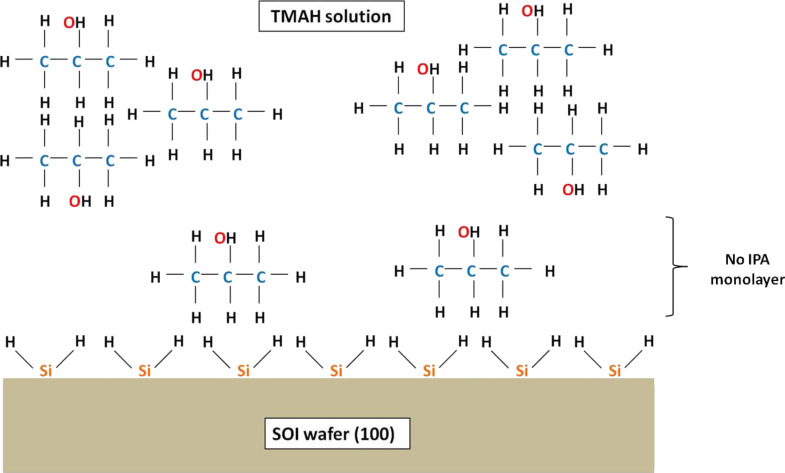
Elimination of the IPA monolayer due to saturation concentration of IPA in TMAH, resulting in a fast etching rate.

The etching rate was calculated by dividing the etching depth by the etching time. Figure 9 shows that the etching rate decreased and reached the lowest value at 20 vol % before increasing sharply at 30 vol %. This phenomenon occurs because the 20 vol % of IPA in 25 wt % TMAH solution is highly concentrated, giving rise to the aggregation of IPA molecules and formation of a monolayer on the Si surface as shown in Figure 10 [28]. This monolayer formation promotes the adsorption of IPA molecules on the Si surface with the hydrocarbon chains binding to the hydrogen-terminated Si surface. In addition, the hydroxyl groups (OH^−^) of IPA are oriented toward the solution. This adsorption would prevent the OH^−^ from etching the Si surface and leads to the reduction in the etching rate. When the IPA concentration was increased to 30 vol %, the IPA and TMAH solutions become saturated, resulting in the disruption of the hydrogen bond network (between IPA molecules and hydrogen-terminated silicon surface) by other excess IPA molecules [28]. This approach would eliminate the monolayer and leads to an increase in the etching rate (Figure 11).

**Figure 12:**
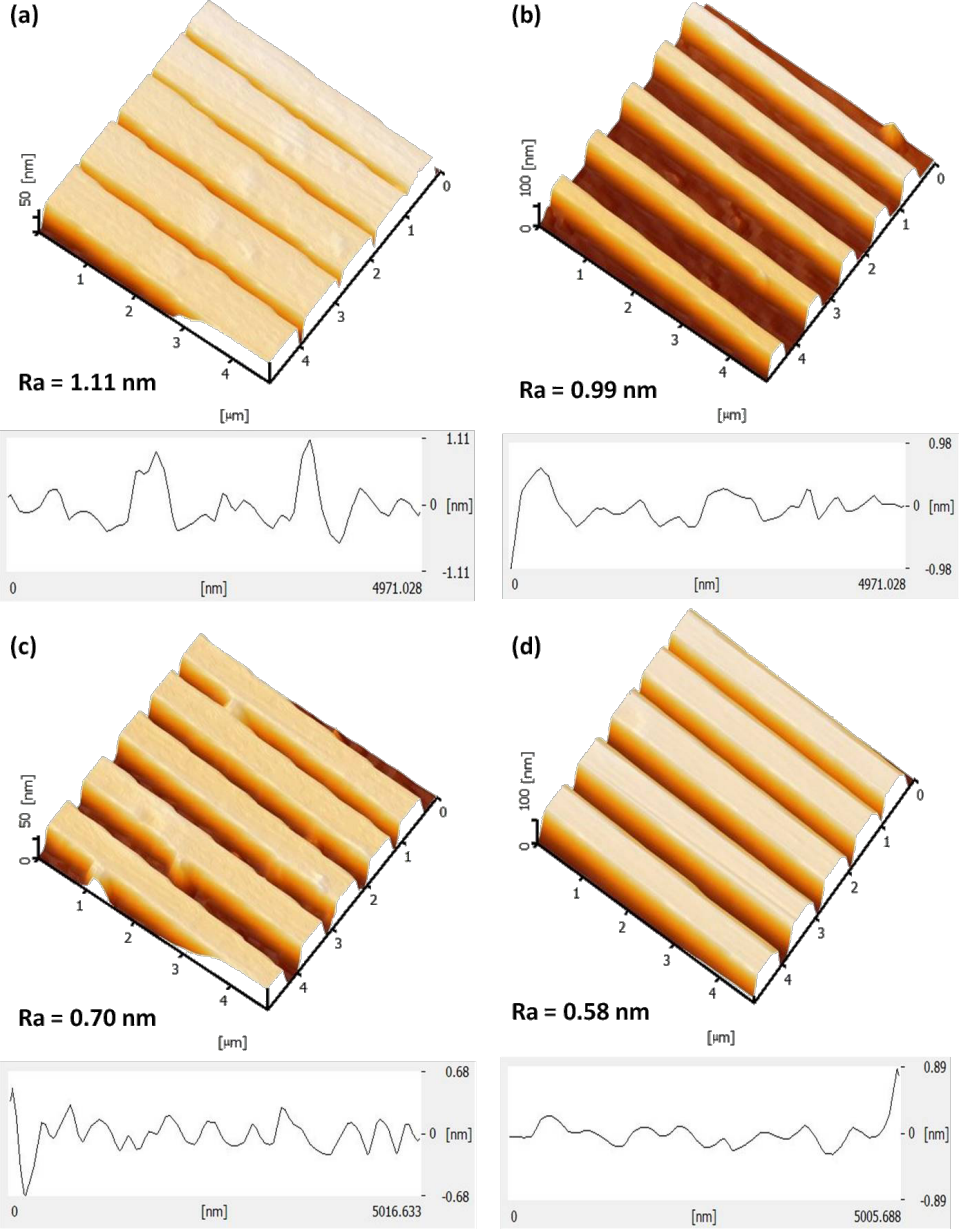
Surface roughness of silicon nanowires by using different concentration of IPA (a) TMAH 25 wt %, without IPA, (b) TMAH 25 wt % and IPA 10 vol %, (c) TMAH 25 wt % and IPA 20 vol %, and (d) TMAH 25 wt % and IPA 30 vol %.

Additionally, the roughness (*R*_a_) values of silicon nanowires were measured using AFM. The surface roughness for particular etching conditions was measured by taking the average profiles of five silicon nanowires. Figure 12 presents the AFM image with *R*_a_ profiles of silicon nanowire etched using TMAH with and without IPA. As shown in Figure 12a, this produced a rougher surface due to formation of hillocks. The existence of hillocks after etching is the main contribution to the rougher surface. When 10 vol % IPA was added to the TMAH solution, the density and size of hillocks becomes smaller (Figure 12b), giving rise to the decrease in the *R*_a_ value. The surface becomes smoother when more IPA was added. It shows that the size of hillocks tend to become small with addition of 20 vol % IPA (Figure 12c) and becomes smaller (Figure 12d) when 30 vol % IPA was used. Hydrogen ions are produced during the wet etching process. This presence of hydrogen can be observed through the generated bubbles coming off the near the SOI surface, influencing the surface roughness [7,29]. The density and size of hillocks is influenced by the hydrogen bubble formation during the etching process. The addition of IPA produces a smooth surface (Figure 9) because it promotes the wettability of the TMAH etchant and decreases the formation of the hydrogen bubbles. Although it is known that a slow etching rate would produce a smooth surface and a fast etching rate produces a rough surface, in this study, it was observed that at 30 vol % IPA, a fast etching rate can still produce a smoother surface due to the reasons discussed above.

**Figure 13:**
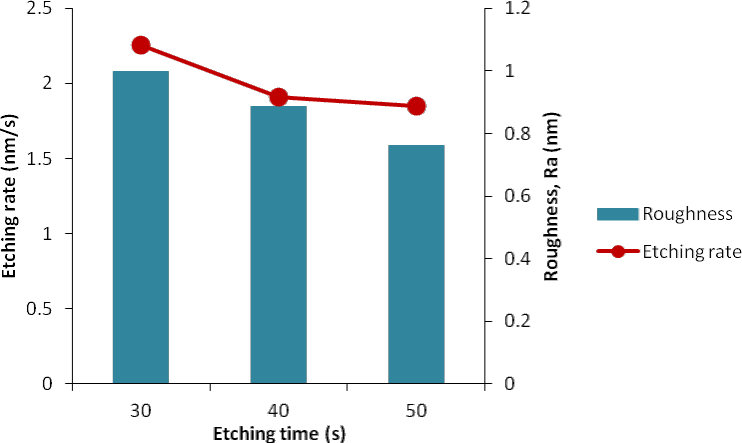
Relationship of etching rate and surface roughness at different etching times.

The etching rate and roughness at various etching times decrease over time with only small differences in the obtained values (Figure 13). This finding explains why the etching rate and surface roughness are not really affected when applying longer etching time at constant concentration of IPA. This small difference occurs due to the small difference of only 10 s between the etching times. However, it is expected that the etching rate and roughness would continue to change for a longer duration of the etching time.

### FESEM and EDX analysis of fabricated silicon nanowires

**Figure 14:**
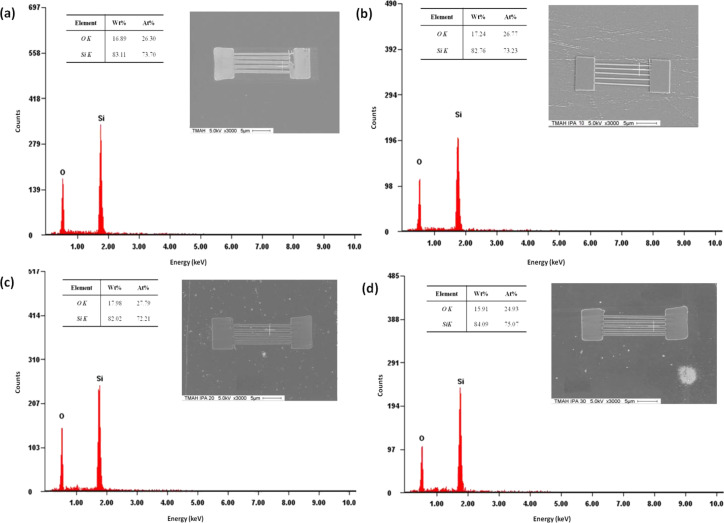
FESEM and EDX analysis of fabricated silicon nanowire structures after etching, (a) TMAH 25 wt %, (b) TMAH 25 wt % and IPA 10 vol %, (c) TMAH 25 wt % and IPA 20 vol %, and (d) TMAH 25 wt % and 30 vol %.

All of the samples underwent FESEM and EDX measurements in order to verify the formation of the silicon nanowires after the etching process. Figure 14 shows Si and O elemental analysis for all cases. The samples etched without IPA and with IPA of 10, 20 and 30 vol % contained 73.7, 73.23, 72.21 and 75.07 atom % of the silicon element, respectively. The pattern etched with 30 vol % IPA shows the highest elemental Si content due to the fast etching rate, resulting in greater removal of the unmasked silicon layer.

Anisotropic silicon wet etching using TMAH is a reduction–oxidation (redox) reaction. The TMAH solution has the molecular structure of (CH_3_)_4_NOH and contains the hydroxide ion (OH^−^) that is useful for etching silicon. The oxidation will occur when hydroxyls react with the silicon surface to form a silicate (Equation 1).

[1]
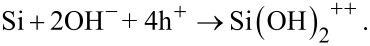


Then, the water reduction reaction occurs to produce hydroxide ions and hydrogen as described by Equation 2. In addition, the silicate will further react with hydroxyls to form a water soluble complex with Si and O as expressed in Equation 3. The overall redox reaction for hydroxide etching by the TMAH solution is shown in Equation 4.

[2]
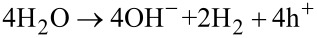


[3]
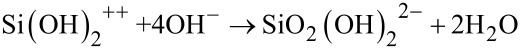


[4]
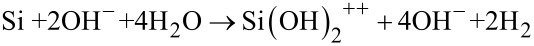


## Conclusion

The thickness and height of a silicon nanowire was controlled by the etching time, and the silicon nanowire width was controlled by the IPA concentration. Furthermore, the surface roughness of the silicon nanowire was influenced by the changes in the IPA concentration in TMAH but did not change considerably with the changes in the etching time. Throughout this study, we observed that reduced etching times would produce thin silicon nanowires and the use of 30 vol % IPA with 25 wt % TMAH produced a smoother surface. This finding can be used to fabricate silicon nanowires with optimal performance for many applications. A further study can be performed to improve the width of silicon nanowires by controlling the width of the oxide mask during AFM lithography.

## Experimental

### Material

In this study, the p-type silicon on an insulating (SOI) wafer with (100) orientation was purchased from SOITEC. It consisted of three layers: the silicon layer (100 nm), the buried oxide layer (200 nm) and the silicon layer (6.25 µm) at the bottom with 13.5–22.5 Ω∙cm resistivity.

### Fabrication of silicon nanowires

An array of silicon nanowires was designed using Nanonavi vector scan software. Then, these patterns were fabricated using AFM lithography (SPI3800N/4000) at a temperature of 24–26 °C with relative humidity of 55–65%. The contact mode, Au cantilever tip (Budgetsensors, Au-coated, ContGB-G) was used at 9 V bias voltage with 0.3 µm/s writing speed. After the AFM lithography process, thin oxide nanowires were formed on the SOI surface functioning as the masking layer for the subsequent etching processes.

### Chemicals

**Figure 15:**
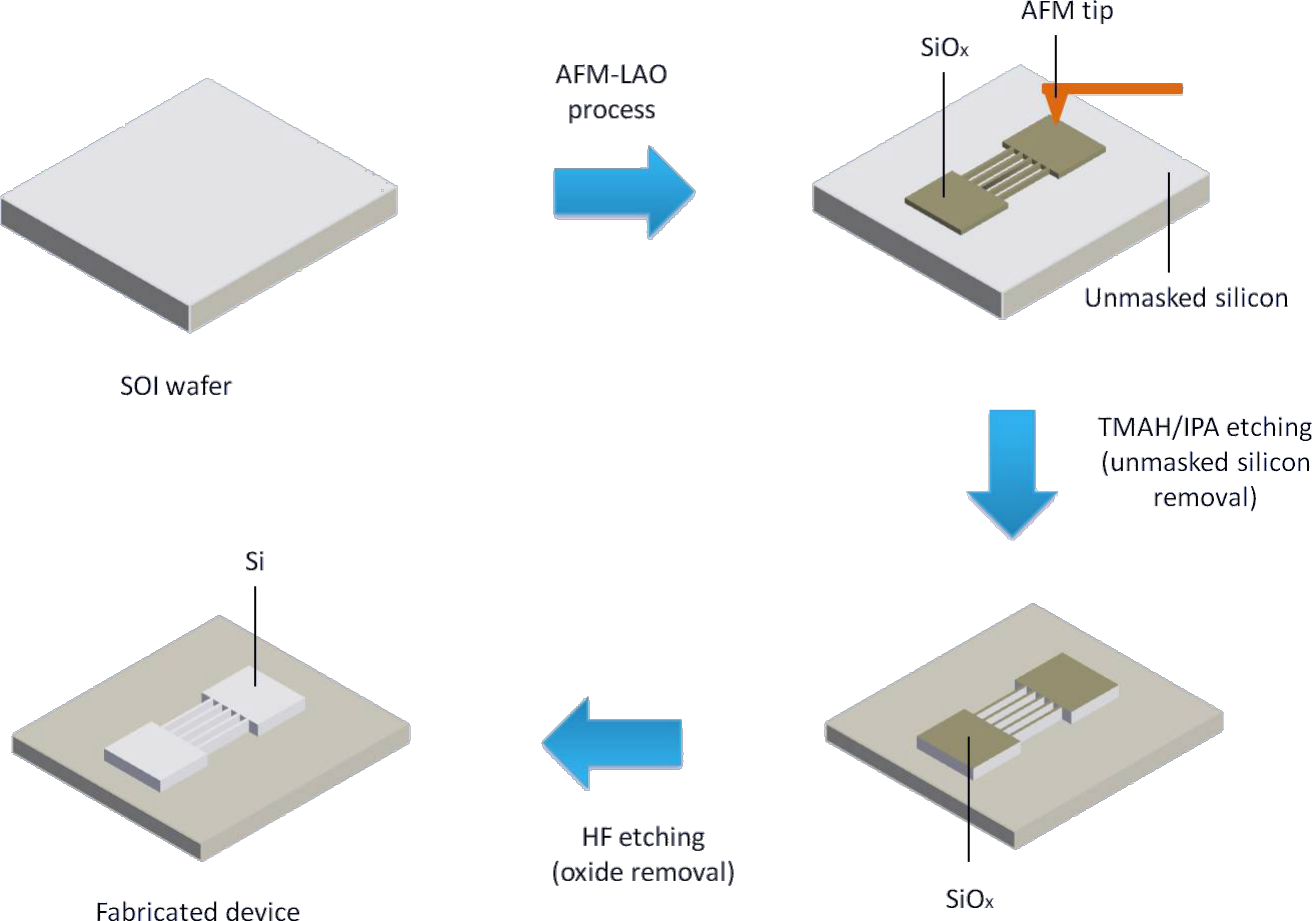
Steps of the silicon nanowire fabrication process.

Tetramethylammonium hydroxide (C_4_H_13_NO) 25 wt % in water and isopropyl alcohol (C_3_H_8_O) were purchased from Sigma-Aldrich. The silicon layers with no oxide layer were removed using 25 wt % TMAH saturated with IPA (10, 20, and 30 vol %) at 65 ºC for 30 s. Then, the masking layers were removed using diluted hydrofluoric acid with deionized water (1:100) for 10 s to obtain the final structure (Figure 15). The experiment was also repeated for different etching times with constant IPA concentration (10 vol %) and temperature (65 °C) in order to study the relationships of the etching depth and width, etching rate and surface roughness.

### Characterization

The surface morphology of the silicon nanowires was characterized in detail using AFM. The elemental analysis of the fabricated silicon nanowire was carried out using FESEM and EDX.
